# Multidrug Resistance in *Pasteurellaceae* Associated With Bovine Respiratory Disease Mortalities in North America From 2011 to 2016

**DOI:** 10.3389/fmicb.2020.606438

**Published:** 2020-11-05

**Authors:** Cassidy L. Klima, Devin B. Holman, Shaun R. Cook, Cheyenne C. Conrad, Brenda J. Ralston, Nick Allan, R. Michele Anholt, Yan D. Niu, Kim Stanford, Sherry J. Hannon, Calvin W. Booker, Tim A. McAllister

**Affiliations:** ^1^Lethbridge Research and Development Centre, Agriculture and Agri-Food Canada, Lethbridge, AB, Canada; ^2^Lacombe Research and Development Centre, Agriculture and Agri-Food Canada, Lacombe, AB, Canada; ^3^Alberta Agriculture and Forestry, Lethbridge, AB, Canada; ^4^Alberta Agriculture and Forestry, Airdrie, AB, Canada; ^5^Chinook Contract Research Inc., Airdrie, AB, Canada; ^6^POV Inc., Airdrie, AB, Canada; ^7^Faculty of Veterinary Medicine, University of Calgary, Calgary, AB, Canada; ^8^Department of Biological Sciences, University of Lethbridge, Lethbridge, AB, Canada; ^9^Feedlot Health Management Services Ltd, Okotoks, AB, Canada

**Keywords:** antimicrobial resistance, bovine respiratory disease, *Mannheimia haemolytica*, *Pasteurella multocida*, integrative and conjugative elements, *Pasteurellaceae*

## Abstract

Multidrug-resistant (MDR; resistance to ≥3 antimicrobial classes) members of the *Pasteurellaceae* family may compromise the efficacy of therapies used to prevent and treat bovine respiratory disease (BRD) in feedlot cattle. This study examined the prevalence of multidrug resistance in strains of *Mannheimia haemolytica* and *Pasteurella multocida* collected from BRD cattle mortalities in North America. Isolates of *M. haemolytica* (*n* = 147) and *P. multocida* (*n* = 70) spanning 69 Alberta feedlots from 2011 to 2016 and two United States feedlots from 2011 to 2012 were examined for antimicrobial resistance (AMR) in association with integrative and conjugative elements (ICEs). Overall, resistance was high in both bacterial species with an increase in the prevalence of MDR isolates between 2011 and 2016. Resistance to >7 antimicrobial drugs occurred in 31% of *M. haemolytica* and 83% of *P. multocida* isolates. Resistance to sulfadimethoxine, trimethoprim/sulfamethoxazole, neomycin, clindamycin oxytetracycline, spectinomycin, tylosin, tilmicosin, and tulathromycin was most common. Although >80% of strains harbored three or more ICE-associated genes, only 12% of *M. haemolytica* and 77% of *P. multocida* contained all six, reflecting the diversity of ICEs. There was evidence of clonal spread as *P. multocida* and *M. haemolytica* isolates with the same pulsed-field gel electrophoresis profile from the United States in 2011 were isolated in Alberta in 2015–2016. This work highlights that MDR strains of *Pasteurellaceae* containing ICEs are widespread and may be contributing to BRD therapy failure in feedlot cattle. Given the antimicrobial resistance gene profiles identified, these MDR isolates may be selected for by the use of macrolides, tetracyclines, and/or in-feed supplements containing heavy metals.

## Introduction

Bovine respiratory disease (BRD) continues to be the principal health problem for beef production, contributing significantly to economic loss through mortality (1% of death loss) and morbidity (up to 80%) (Hilton, 2014). Multiple viral [bovine herpesvirus type 1 (BHV-1), parainfluenza-3 virus (PI3), bovine viral diarrhea virus (BVDV), and bovine respiratory syncytial virus (BRSV)] and bacterial agents (*Mannheimia haemolytica, Pasteurella multocida, Histophilus somni*, and *Mycoplasma bovis*) are implicated in BRD, resulting in a disease complex comprised of numerous forms of pneumonia with varied pathologies. These include bronchopneumonia and fibrinous pneumonia typically associated with *P*. *multocida* and *M*. *haemolytica*, respectively (Booker et al., 2008); both species are members of the *Pasteurellaceae* family.

Because it is a complex infection, prevention and management of BRD is challenging. While vaccination is used to protect against viral infections, antimicrobials are the primary tool used to address bacterial agents. As a result, attempts to prevent or treat BRD contribute significantly to the volume of antimicrobials used in beef production. Frequently this is in the form of injectable metaphylaxis used in cattle at high risk of developing respiratory infection upon arrival at feedlots. However, targeted antimicrobial treatment is also used for cattle diagnosed with any ailment due to bacterial infection, which can include cattle exhibiting symptoms of respiratory illness. Additionally, frequent in-feed use of antimicrobials like chlortetracycline and tylosin to prevent or reduce the incidence of liver abscesses can also contribute to bacterial drug exposure in feedlots. Although the use of antimicrobials in the beef industry is necessary to support production and animal health it carries an associated risk of promoting the development and/or selection of antimicrobial-resistant bacteria. This selection pressure may be higher for pathogenic bacteria that are the primary targets of antimicrobial therapy, such as the BRD-associated species, as they are likely to be more frequently exposed to drugs than commensal species.

Multidrug resistance development in bacterial pathogens is a growing concern as microbial agents resistant to multiple drugs can be co-selected by the use of different drug classes. Interim standard definitions (Kwon et al., 2012) have defined multidrug-resistant strains as those resistant to at least one agent in three or more antimicrobial categories and extensively drug-resistant (XDR) strains as those non-susceptible to at least one agent in all but two or fewer antimicrobial categories. For the purpose of this dataset we identify strains as XDR when they are resistant to greater than seven of the nine antimicrobial classes tested, although we did not test all possible drug classes within this panel.

Since the report of a MDR strain of *P. multocida* harboring 12 antimicrobial resistance genes (ARGs) in Michael et al. (2012a), multidrug resistance has been repeatedly documented in *Pasteurellaceae* isolates recovered from cattle (Michael et al., 2012a; Lubbers and Hanzlicek, 2013; Klima et al., 2016; Rainbolt et al., 2016; Snyder et al., 2017). Many of the antimicrobial ARGs in these isolates are associated with integrative and conjugative elements (ICEs) (Michael et al., 2012a, b; Eidam et al., 2015; Klima et al., 2016). ICEs are self-transmissible, mobile genetic elements (MGEs) that can contain cassettes of accessory genes enhancing host survivability, including genes associated with environmental adaptation (e.g., ARGs, heavy metal resistance, or phage resistance genes), metabolism (sucrose degradation genes), and fitness (e.g., bacteriocin, nitrogen fixation, biofilm formation, and DNA repair genes) (Armshaw and Pembroke, 2013). Although ICE transfer can be similar to that of plasmids, they differ in that they integrate into the host chromosome and are maintained during cell division (Johnson and Grossman, 2015). Notably, ICEs identified in *Pasteurellaceae* isolates contain toxin-antitoxin systems that promote their persistence in bacterial populations irrespective of any selective pressure in their environment (Klima et al., 2016).

The ability of ICEs to accumulate and transfer ARGs between *Pasteurellaceae* spp. may promote the spread of antimicrobial resistance (AMR) in beef cattle production. To address the potential development and dissemination of MDR BRD pathogens within the North American cattle production system, a better understanding of the prevalence of multidrug resistance and its association with ICE is required. This study examines MDR strains of *M. haemolytica* and *P. multocida* originating from BRD cattle mortalities as part of three longitudinal studies spanning 2011–2016. The purpose was to describe the diversity of multidrug resistance found in these BRD isolates collected over geographic and temporal distances, and to examine genetic associations between observed multidrug resistance and existing ICEs previously described in these species.

## Materials and Methods

### Sample Collection, Isolation, and Antimicrobial Susceptibility Testing of *M. haemolytica* and *P. multocida*

Samples used for this study originated from three longitudinal projects that collected post-mortem lung tissue samples (*n* = 755) from cattle BRD mortalities between 2011–2012 (study 1), 2014–2015 (study 2), and 2015–2016 (study 3) (Table 1). Deceased animals were identified, and samples collected based on gross pathological evidence of infectious pneumonia. Infected tissues were excised aseptically from the perimeter of pneumonic lesions, collected in sterile containers, and transported to diagnostic laboratories for processing. Samples from study 1 consisted of fresh tissue stored at 4°C for a period no longer than 10 days prior to bacterial isolation (Klima et al., 2014b). In studies 2 and 3, when delivery time was projected to exceed 72 h, tissues were frozen and stored at −20°C until processed (Anholt et al., 2017; Stanford et al., 2020). Metadata for the animals sampled were provided from submitting veterinary practices and included diagnosis and gross pathology for each case. Drug use data, in the form of how many treatments each animal received, was provided from all veterinary practices and all but one practice also specified the specific antimicrobials used. In total, seven of the 33 feedlots sampled were represented in more than one study (i.e., each were represented in both study 2 and study 3) (Supplementary Table S1).

**TABLE 1:** Characteristics of each longitudinal study; the number of isolates screened using antimicrobial susceptibility testing (AST), percentage of multidrug-resistant (MDR) isolates detected, and number of isolates from each study used for subsequent analyses.

	Surveillance study 1	Surveillance study 2	Surveillance study 3	Total
Year(s) study was performed	2011–2012	2014–2015	2015–2016	–
**No. of feedlots sampled^a^**				
Canada	10	17	12	31
United States	2	0	0	2
**No. animals sampled in the study**	**68**	**528**	**159**	**755**
**No. of isolates recovered**				
*M. haemolytica*	55	238	35	408
*P. multocida*	8	85	34	140
**Percentage of isolates found to be MDR^b^ (No. of strains screened with AST)**				
*M. haemolytica*	67.3 (55)	55 (238)	81.8 (33)	–
*P. multocida*	50 (8)	70.5 (85)	90.9 (33)	–
**No. of MDR isolates included in this analysis**				
*M. haemolytica*	40	80	27	147
*P. multocida*	6	34	30	70
Total	46	114	57	217

*^a^Values include feedlots where MDR strains were recovered. Only three feedlots were represented in more than one study, both sampled in study 2 and study 3 (Supplementary Table S1).^b^MDR = an isolate expressing resistance to ≥3 antimicrobial classes.*

All lung tissue samples were processed for the isolation of *M. haemolytica* and *P. multocida* and tested for antimicrobial susceptibility with broth microdilution using a commercially available bovine/porcine BOPO6F panel (Sensititre; Trek Diagnostic Systems, Cleveland, OH, United States) as previously described for *M. haemolytica* (Klima et al., 2014a) and as per the manufacturer for *P. multocida*. The minimum inhibitory concentrations (MICs) were assigned as outlined in the Clinical and Laboratory Standards document M31-A3 (Clinical and Laboratory Standards Institute [CLSI], 2008) for those antimicrobials with described breakpoints. Antimicrobials that lacked breakpoints were not assigned a susceptibility designation, with the exception of those antimicrobials where populations exhibited a bimodal distribution with high MICs and isolates possessed the corresponding resistant determinant (Supplementary Table S2). Methods used for species identification and serotyping are detailed in Klima et al. (2014b). Isolates expressing resistance to three or more antimicrobial drugs were classified as MDR and selected for further analysis in this study. Longitudinal study 1 incorporated Canadian and American feedlot sites, while studies 2 and 3 only included feedlots in Canada. The number of feedlots sampled, the number of strains isolated per bacterial species, the percentage of multidrug resistance observed in each study, and the number of MDR isolates included in this analysis are listed in Table 1. In total, 147 *M. haemolytica* and 70 *P. multocida* isolates were genotyped using pulsed-field gel electrophoresis (PFGE) with 95% similarity as a cut-off as per Klima et al. (2011) and ICE-associated genes and ARGs screened using PCR as described below.

### PCR Assays

Three multiplex PCR assays were designed to improve the efficiency of ARG screening. Primers were designed to target genes in ICE*Pmu1* (Michael et al., 2012b) using MPprimer v.1.5 (Shen et al., 2010). The RR1-MPLEX assay targeted five ARGs (*floR*, *strB*, *aphA1*, *strA*, and *sul2*) in an accessory gene region of ICE*Pmu1* termed resistance region 1. The RR2-MPLEX assay amplified four ARGs [*tet*(H), *bla*_OXA–2_, *aadA25*, and *aadB*] in an accessory gene region of ICE*Pmu1* termed resistance region 2 as well as a multicopper oxidase gene (*mco*) located between resistance region 1 and 2. The MCRLD-MPLEX targeted three macrolide ARGs [*msr*(E), *mph*(E), and *erm*(42)] dispersed across both ICE*Pmu1* regions 1 and 2. Five genes that are part of the core functional regions of ICEPmu1, including a hypothetical protein gene (Pmu_02680), an integrase gene (Pmu_02700), a relaxase gene (Pmu_02890), a transposase gene (Pmu_03510), and a binding protein gene (Pmu_03540), were also screened for by individual PCR assays as previously described (Klima et al., 2014b).

### Multiplex PCR Conditions

**FIGURE 1:**
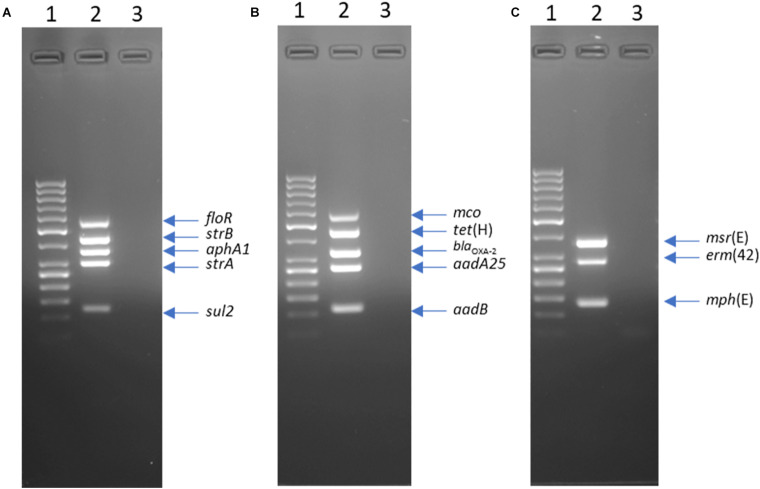
Agarose gel electrophoresis of products from multiplex PCR RR1-MPLEX **(A)** RR2-MPLEX, **(B)** and MCRLD-MPLEX **(C)**. Lanes: 1, GeneRuler 50 bp DNA Ladder; 2, Mannheimia haemolytica strain L024A (accession LFXX00000000); 3, negative control.

Template DNA was generated using a 1 μL loop of bacterial culture that was suspended in 100 μL of TE buffer (10 mM Tris–HCl, 1 mM EDTA; pH 8.00) and lysed at 98°C for 5 min. The lysate was centrifuged at 13,000 × *g* for 5 min and 2 μL of supernatant was used as template DNA. For RR1-MPLEX, RR2-MPLEX, and MCRLD-MPLEX, PCR mixtures contained 1X Qiagen Multiplex PCR Master Mix (Qiagen Inc., Mississauga, ON, Canada), 0.5 × Qiagen Q-Solution, varying concentrations of each associated primer as listed in Supplementary Table S3, and 2 μL of template DNA in a total volume of 50 μL. All PCRs were performed as follows: 95°C for 15 min; 35 cycles of 94°C for 30 s, 62°C for 45 s, and 72°C for 1 min; with a final extension at 72°C for 10 min. Following PCR, 20 μL of product was resolved by electrophoresis in a 2.0% (w/v) agarose gel alongside a 50 bp DNA ladder (GeneRuler; Thermo Fisher Scientific, Burlington, ON, Canada) and visualized using ethidium bromide (Figure 1). Representative PCR products from 12 *M*. *haemolytica* and *P*. *multocida* isolates were sequenced to confirm the accuracy of amplified fragments.

### Statistical Analysis

Fisher’s exact test and Cramer’s V were used in R v. 3.5.1 with the vcd package v. 4.1–4 to determine the strength of associations between categorical (nominal) variables for MDR *M*. *haemolytica* and *P*. *multocida* isolates. Multidrug resistance profile, study, feedlot, country, submitting veterinary practice, diagnosis, profile of antimicrobial treatment, individual drug treatment, individual resistance phenotypes, and ARG presence were included in the analysis. Associations between the presence/absence of ARGs and antimicrobial resistance phenotype in *M*. *haemolytica* and *P*. *multocida* were assessed using Pearson’s correlation coefficient in R. All *P*-values were corrected for multiple comparisons using the Benjamini-Hochberg method. Cramer’s *V* values close to 0 indicate little association between variables while those close to 1 indicate a strong association.

## Results and Discussion

### Antimicrobial Susceptibility Testing of *M*. *haemolytica* and *P*. *multocida* Isolates

**FIGURE 2:**
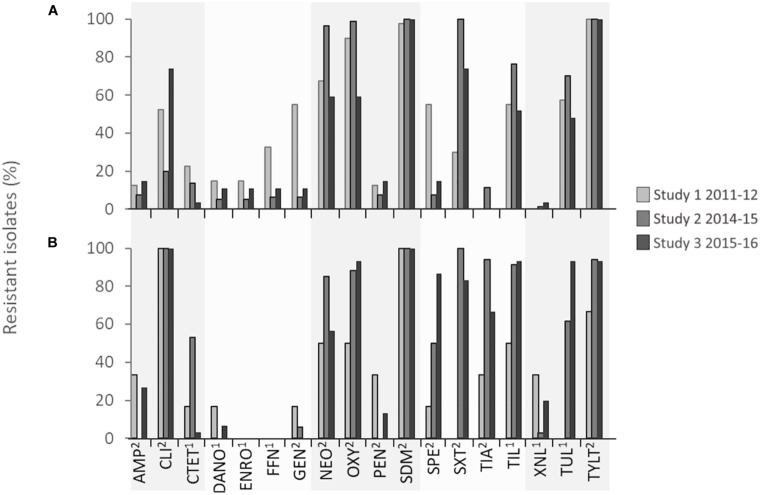
Percentage of **(A)** *Mannheimia haemolytica* and **(B)** *Pasteurella multocida* isolates in each surveillance study that were resistant to each antimicrobial. ^1^Minimum inhibitory concentration (MIC) breakpoints defined by Clinical Laboratory Standards Institute (CLSI) guidelines. ^2^MIC determined by bimodal distribution of MIC coupled with the presence of associated antimicrobial resistance genes. ^2^Ampicillin, AMP; ^2^Clindamycin, CLI; ^1^Chlortetracycline, CTET; ^1^Danofloxacin, DANO; ^1^Enrofloxacin, ENRO; ^1^Florfenicol, ^2^FFN; Gentamicin, GEN; ^2^Neomycin, NEO; ^2^Oxytetracycline, OXY; ^2^Penicillin, PEN; ^2^Trimethoprim/sulfamethoxazole, SDM; ^2^Spectinomycin, SPE; ^2^Sulfadimethoxine, SXT; ^2^Tiamulin, TIA; ^1^Tilmicosin, TIL; ^1^Ceftiofur, XNL; ^1^Tulathromycin, TUL; ^2^Tylosin tartrate, TYLT.

*Pasteurella multocida* isolates showed very high levels of resistance (>75% of isolates within each study) to neomycin, oxytetracycline, spectinomycin, tiamulin, tilmicosin, tulathromycin, tylosin, and trimethoprim/sulfamethoxazole, and complete resistance (100% of isolates) to clindamycin and sulfadimethoxine in all three studies (Figure 2 and Table 2). With the exception of clindamycin and sulfadimethoxine, the frequency of drug resistance fluctuated over time, but an increase in the percentage of isolates resistant to oxytetracycline, spectinomycin, tilmicosin, and tulathromycin occurred from 2011 to 2016. *M. haemolytica* isolates were completely resistant (100% of isolates) to tylosin in all years and to sulfadimethoxine from 2014 to 2016. Overall, high levels of resistance (>75% of isolates within a study) to neomycin, oxytetracycline, trimethoprim/sulfamethoxazole, and sulfadimethoxine were observed in *M. haemolytica* isolates. However, this varied by year, with the highest levels of resistance to florfenicol, gentamicin, and spectinomycin observed in 2011–2012 and to neomycin, oxytetracycline, trimethoprim/sulfamethoxazole, and macrolides in 2014–2015. Although a trend toward higher resistance to some antimicrobials was observed over time in both *P. multocida* and *M. haemolytica* isolates, there was considerable temporal and/or geographical variation in resistance to most antimicrobials. Of the antimicrobials included for susceptibility testing, only macrolides (tilmicosin, tulathromycin, tylosin), tetracyclines (chlortetracycline, oxytetracycline), β-lactams (ceftiofur), phenicols (florfenicol), and fluoroquinolones (danofloxacin, enrofloxacin) were used frequently in feedlots (Brault et al., 2019). As a result, the resistance observed to aminoglycosides (gentamicin, neomycin, spectinomycin), lincosamides (clindamycin), and pleuromutilins (tiamulin) was unexpected.

**TABLE 2:** Multidrug resistance profiles and associated pulsotypes for *Mannheimia haemolytica* and *Pasteurella multocida* isolates collected from bovine respiratory disease mortalities.

No. drugs in MDR profile	No. drug classes isolates are resistant to	Drugs isolates are resistant to	Pulsotype	*M. haemolytica*	*P. multocida*
3	3	CLI, OXY, SDM			
			59		1
			61		1
3	3	CLI, SDM, TIA			
			57		1
3	3	CLI, SDM, TYLT			
			5	1	
			9	1	
			19	1	
			38	1	
			49	1	
3	3	OXY, SDM, TYLT			
			25	1	
			26	1	
4	3	CLI, NEO, SDM, SXT			
			61		1
	3	CLI, SDM, SXT, TYLT			
			4	1	
			15	1	
			26	1	
			27	1	
			29	1	
			37	1	
			42	2	
			45	1	
4	4	NEO, OXY, SDM, TYLT			
			1	1	
			12	2	
			21	2	
			26	1	
			29	1	
			39	1	
4	4	CLI, SDM, TIA, TYLT			
			58		1
4	3	AMP, CLI, PEN, SDM, XNL			
			55		1
4	3	CLI, GEN, NEO, SDM, SXT			
			63		1
4	5	CLI, NEO, OXY, SDM, TYLT			
			35	1	
4	4	CLI, SDM, TIL, TUL, TYLT			
			61		1
4	4	NEO, OXY, SDM, SXT, TYLT			
			2	1	
			3	1	
			21	7	
			22	1	
			29	1	
			32	3	
			36	1	
6	5	CLI, NEO, OXY, SDM, SXT, TYLT			
			21	1	
			22	1	
			32	2	
6	5	CLI, NEO, OXY, SDM, TIL, TYLT			
			57		1
6	4	FFN, NEO, SDM, SXT, TUL, TYLT			
			15	1	
6	4	NEO, OXY, SDM, SXT, TIL, TYLT			
			21	2	
			30	1	
6	4	NEO, OXY, SDM, TIL, TUL, TYLT			
			21	1	
6	3	OXY, SDM, SXT, TIL, TUL, TYLT			
			23	3	
7	5	CLI, CTET, NEO, OXY, SDM, SXT, TYLT			
			21	1	
7	5	CLI, CTET, NEO, OXY, SDM, TIL, TYLT			
			54		1
7	4	CLI, CTET, OXY, SDM, TIL, TUL, TYLT			
			61		1
7	6	CLI, FFN, NEO, OXY, SDM, TIL, TYLT			
			12	1	
7	6	CLI, NEO, OXY, SDM, SXT, TIA, TYLT			
			26	1	
7	5	CLI, NEO, OXY, SDM, SXT, TIL, TYLT			
			21	1	
			26	1	
7	4	CTET, GEN, OXY, SDM, SPE, TUL, TYLT			
			12	1	
7	3	CTET, GEN, OXY, SPE, TIL, TUL, TYLT			
			12	1	
7	4	NEO, OXY, SDM, SPE, SXT, TIL, TYLT			
			32	1	
8	4	NEO, OXY, SDM, SXT, TIL, TUL, TYLT			
			6	1	
			8	1	
			15	1	
			17	1	
			21	20	
			22	1	
			24	1	
			26	1	
			28	1	
			29	1	
			32	8	
			33	1	
			34	1	
			40	1	
			53	1	
8	5	CLI, NEO, OXY, SDM, TIL, TUL, TYLT			
			21	1	
			61		1
8	5	CLI, NEO, OXY, SDM, SPE, TIL, TUL, TYLT		1	
			61		
8	5	CLI, NEO, OXY, SDM, SXT, TIL, TUL, TYLT			
			16	1	
			26	1	
			32	2	
8	5	CLI, NEO, SDM, SXT, TIA, TIL, TUL, TYLT			
			61		1
8	5	CLI, OXY, SDM, SPE, SXT, TIL, TUL, TYLT			
			61		2
8	6	CLI, OXY, SDM, SPE, TIA, TIL, TUL, TYLT			
			61		1
8	5	CLI, OXY, SDM, SXT, TIA, TIL, TUL, TYLT			
			61		1
8	5	CTET, GEN, OXY, SDM, SPE, SXT, TUL, TYLT			
			48	1	
8	4	CTET, NEO, OXY, SDM, SXT, TIL, TUL, TYLT			
			21	2	
8	4	NEO, OXY, SDM, SPE, SXT, TIL, TUL, TYLT			
			15	1	
8	5	NEO, OXY, SDM, SXT, TIA, TIL, TUL, TYLT			
			21	1	
			32	2	
9	6	AMP, CLI, NEO, OXY, PEN, SDM, SXT, TIL, TYLT			
			21	1	
9	6	AMP, CLI, NEO, OXY, PEN, SDM, SXT, XNL, TYLT			
			20	1	
9	6	CLI, CTET, NEO, OXY, SDM, SXT, TIA, TIL, TYLT			
			31	1	
			61		1
			62		1
			64		1
			65		1
			68		1
			70		1
			72		2
			74		1
9	6	CLI, CTET, NEO, SDM, SXT, TIA, TIL, TUL, TYLT			
			61		1
9	5	CLI, CTET, OXY, SDM, SXT, TIA, TIL, TUL, TYLT			
			61		1
9	5	CLI, NEO, OXY, SDM, SPE, SXT, TIL, TUL, TYLT			
			61		1
9	6	CLI, OXY, SDM, SPE, SXT, TIA, TIL, TUL, TYLT			
			61		4
9	6	CTET, NEO, OXY, SDM, SXT, TIA, TIL, TUL, TYLT			
			21	1	
			32	1	
9	5	DANO, ENRO, GEN, OXY, SDM, SPE, SXT, TUL, TYLT			
			14	1	
10	6	AMP, CLI, NEO, OXY, SDM, SPE, SXT, TIL, TUL, TYLT			
			61		2
10	5	CLI, CTET, GEN, NEO, OXY, SDM, SPE, TIL, TUL, TYLT			
			52	1	
10	6	CLI, CTET, NEO, OXY, SDM, SXT, TIA, TIL, TUL, TYLT			
			21	1	
			61		1
10	6	CLI, CTET, OXY, SDM, SPE, SXT, TIA, TIL, TUL, TYLT			
			61		1
10	6	CLI, FFN, GEN, NEO, OXY, SDM, SPE, TIL, TUL, TYLT			
			12	2	
			32	1	
			41	1	
			48	1	
10	6	CLI, NEO, OXY, SDM, SPE, SXT, TIA, TIL, TUL, TYLT			
			21	1	
			61		10
			62		1
			66		1
			69		1
			70		1
			72		3
			73		1
10	7	CLI, OXY, SDM, SPE, SXT, TIA, TIL, XNL, TUL, TYLT			
			61		1
11	7	AMP, CLI, DANO, OXY, SDM, SPE, SXT, TIA, TIL, TUL, TYLT			
			61		1
11	6	AMP, DANO, ENRO, GEN, OXY, PEN, SDM, SPE, TIL, TUL, TYLT			
			13	1	
			39	1	
11	6	CLI, CTET, FFN, GEN, NEO, OXY, SDM, SPE, TIL, TUL, TYLT			
			7	1	
			26	1	
			46	1	
11	6	CLI, CTET, GEN, NEO, OXY, SDM, SPE, SXT, TIL, TUL, TYLT			
			12	1	
11	7	CLI, CTET, GEN, OXY, SDM, SPE, SXT, TIA, TIL, XNL, TYLT			
			61		1
11	6	CLI, CTET, NEO, OXY, SDM, SPE, SXT, TIA, TIL, TUL, TYLT			
			61		1
			62		1
			67		1
			71		1
11	6	CLI, FFN, GEN, NEO, OXY, SDM, SPE, SXT, TIL, TUL, TYLT			
			12	1	
			15	1	
			47	1	
11	7	CLI, NEO, OXY, SDM, SPE, SXT, TIA, TIL, XNL, TUL, TYLT			
			61		1
12	7	AMP, CLI, DANO, GEN, NEO, OXY, PEN, SDM, SPE, TIL, XNL, TYLT			
			56		1
12	8	AMP, CLI, DANO, OXY, PEN, SDM, SPE, SXT, TIA, TIL, TUL, TYLT			
			61		1
12	7	AMP, CLI, NEO, OXY, SDM, SPE, SXT, TIA, TIL, XNL, TUL, TYLT			
			61		1
12	6	AMP, CTET, DANO, ENRO, GEN, OXY, PEN, SDM, SPE, TIL, TUL, TYLT			
			18	1	
13	6	AMP, CLI, CTET, GEN, NEO, OXY, PEN, SDM, SXT, TIL, XNL, TUL, TYLT			
			32	1	
13	7	AMP, CLI, DANO, ENRO, GEN, OXY, PEN, SDM, SPE, SXT, TIL, TUL, TYLT			
			18	1	
13	7	AMP, CLI, NEO, OXY, PEN, SDM, SPE, SXT, TIA, TIL, XNL, TUL, TYLT			
			60		1
			61		2
15	8	AMP, CLI, CTET, DANO, ENRO, FFN, GEN, NEO, OXY, PEN, SDM, SPE, TIL, TUL, TYLT			
			10	1	
15	8	AMP, CLI, DANO, ENRO, FFN, GEN, NEO, OXY, PEN, SDM, SPE, SXT, TIL, TUL, TYLT			
			7	1	
			11	1	
			26	1	
			32	1	
16	8	AMP, CLI, CTET, DANO, ENRO, FFN, GEN, NEO, OXY, PEN, SDM, SPE, SXT, TIL, TUL, TYLT			
			21	1	
			32	1	
			50	1	
			Total	147	70

The markedly higher resistance to clindamycin, tiamulin, and ceftiofur in *P. multocida* isolates compared to *M. haemolytica* is difficult to explain (Figure 2). Although not frequently administered to cattle, clindamycin and tiamulin use is common in swine production where *P. multocida* is an important cause of pneumonia and atrophic rhinitis (Davies et al., 2003). Consequently, it is possible that these phenotypes could originate from other food production sectors where *P. multocida* is a pathogen frequently targeted with these therapeutics.

There has been recent emergence of pleuromutilin (tiamulin) and lincosamide (clindamycin) resistance in *Brachyspira hyodysenteriae*, an agent of swine dysentery worldwide that has been associated with *lnu(*C) (lincosamide resistance), *rplC* and *tva*(A) (pleuromutilin resistance), and various point mutations in the 23S rRNA gene (macrolide, lincosamide, and pleuromutilin resistance) (Card et al., 2018). These mechanisms have not been examined here, but cross-resistance has been documented within the MLS_B_ (macrolides, lincosamides and streptogramins B) group of antimicrobials, with the rRNA methylase gene *erm*(42) shown to confer strong resistance to clindamycin in *P. multocida in vitro* (Desmolaize et al., 2011). Correlation between clindamycin and macrolide resistance in *P. multocida* isolates in the present study was not 100% as six clindamycin-resistant isolates were susceptible to all macrolides tested. Likewise, five tiamulin-resistant isolates were susceptible to clindamycin indicating that further characterization is needed to determine what is contributing to the resistance observed.

Why moderate levels of resistance to ceftiofur (<30% of isolates) were observed in *P. multocida* isolates, but not in *M. haemolytica*, is also unclear as this antimicrobial is used in both beef and pork production for the treatment and control of respiratory infections. Historically, in Canada, ceftiofur (a third-generation cephalosporin) has been used less frequently than tetracyclines, florfenicol, or macrolides to treat BRD-associated infections in cattle (Brault et al., 2019). Consequently, ceftiofur is still potentially highly efficacious against BRD agents with <1% of *Pasteurellaceae* isolates collected from the United States and Canada expressing resistance (Klima et al., 2014b; Anholt et al., 2017; Timsit et al., 2017; Kadlec et al., 2018; Woolums et al., 2018). However, recent increased resistance in *Pasteurellaceae* spp. to the previously mentioned and more commonly used drugs (Klima et al., 2014b; Anholt et al., 2017; Timsit et al., 2017; Kadlec et al., 2018; Woolums et al., 2018), may lead to an increase in the employment of ceftiofur for BRD therapy. This is concerning, because in addition to being an important veterinary therapy, ceftiofur is classified in Canada as an antimicrobial of very high importance in human medicine (category I) (Government of Canada, 2009) and can select for extended-spectrum β-lactamase-producing *Enterobacteriaceae* (Mollenkopf et al., 2017). Therefore, the development of cross-resistance to other cephalosporins because of ceftiofur use in veterinary medicine could potentially negatively affect human therapies.

Interestingly, although some florfenicol resistance was observed in *M. haemolytica*, no resistance to this drug was seen in *P. multocida.* Similar to tiamulin, florfenicol is licensed for use in both swine and cattle for the treatment of bacterial pneumonia, although it may be used more frequently in cattle with only 7 to 13% of swine farms in Canada reporting injectable florfenicol use in growing-finishing pigs between 2013 and 2016 (Government of Canada, 2018).

### Multidrug Resistance in *M*. *haemolytica* and *P*. *multocida*

**FIGURE 3:**
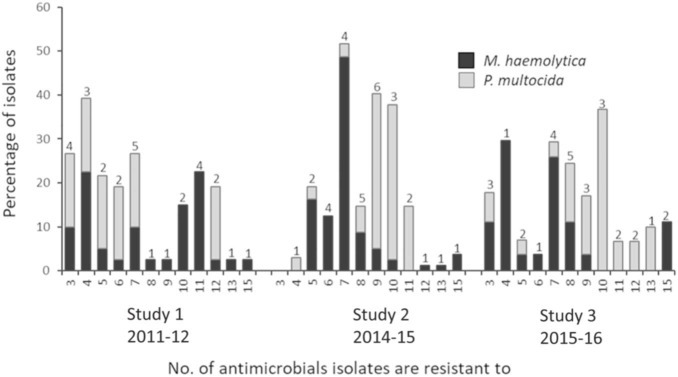
Multidrug resistance in *Mannheimia haemolytica and Pasteurella multocida* isolates. Numbers above bars indicate the number of unique antimicrobial susceptibility testing profiles within each multidrug resistance category.

Possible XDR *M. haemolytica* and *P. multocida* isolates were found in all three studies (Figure 3 and Table 2). Of the 104 unique multidrug resistance profiles identified across both species, three *M. haemolytica* isolates from two different feedlots in Canada, were resistant to 16 antimicrobials spanning nine antimicrobial classes (ampicillin, clindamycin, chlortetracycline, danofloxacin, enrofloxacin, florfenicol, gentamicin, neomycin, oxytetracycline, penicillin, sulfadimethoxine, spectinomycin, tilmicosin, trimethoprim/sulfamethoxazole, tulathromycin, and tylosin). From 2011 to 2016 the prevalence of *M. haemolytica* isolates resistant to >7 antimicrobials fell from 50 to 26%. In contrast, the incidence of *P. multocida* isolates resistant to >7 antimicrobials increased from 17% in 2011 to 87% in 2016. It is unclear why there was an increase in the number of *P. multocida* resistant to a large number of drugs, but not in *M. haemolytica*; particularly since the overall level of multidrug resistance in *M. haemolytica* was highest at 86% in 2016. The variability in the AMR profiles and the lack of correlation of these profiles with genotype (see below) suggests that horizontal gene transfer rather than clonal dissemination is contributing to the complexity of multidrug resistance in *Pasteurellaceae* spp. in cattle. It is possible that the rate of transfer and overall stability of the ARGs present in *M. haemolytica* ICEs could differ from that of *P. multocida*, accounting for some of the variability in prevalence and AMR patterns seen between the species. Some ICEs have been shown to be active in the host immediately before becoming quiescent. Having not integrated directly into the host genome, these elements are not yet under repression from their ICE-encoded regulators and therefore can rapidly spread to other bacterial cells (Johnson and Grossman, 2015). Although speculation at this point, it is possible that the kinetics behind the transfer and integration rates of ICEs could differ among species.

### Co-Isolation of *M. haemolytica* and *P. multocida*

Of the 946 lung tissue samples collected, only 20 animals were culture positive for both MDR *M. haemolytica* and *P. multocida*. Of these cases, two animals harbored isolates of *M. haemolytica* and *P. multocida* that exhibited identical multidrug resistance profiles, with one pair of isolates resistant to clindamycin, chlortetracycline, neomycin, oxytetracycline, sulfadimethoxine, trimethoprim/sulfamethoxazole, tiamulin, tilmicosin, tylosin and the other resistant to clindamycin, neomycin, oxytetracycline, sulfadimethoxine, spectinomycin, trimethoprim/sulfamethoxazole, tiamulin, tilmicosin, tulathromycin, and tylosin. The remaining 18 animals with co-infections harbored strains of *M. haemolytica* and *P. multocida* that had different multidrug resistance profiles.

### Detection of ICE and Antimicrobial Resistance Genes in *M. haemolytica* and *P. multocida*

Since the discovery of ICE*Pmu1* in *P. multocida* (Michael et al., 2012a), and ICE*Mh1* in *M. haemolytica* (Eidam et al., 2015), multiple ICEs have been documented in *Pasteurellaceae*, many of which are associated with possible XDR (Klima et al., 2016; Cameron et al., 2018; Kadlec et al., 2018; Stanford et al., 2020). All have a similar backbone with regions of functional genes associated with conjugation, recombination and regulation grouped together and in similar orientation. As a result, much of the diversity observed occurs due to the presence or absence, and varied composition, of two accessory gene cassettes that can harbor ARGs, heavy metal resistance genes and other virulence factors.

**FIGURE 4:**
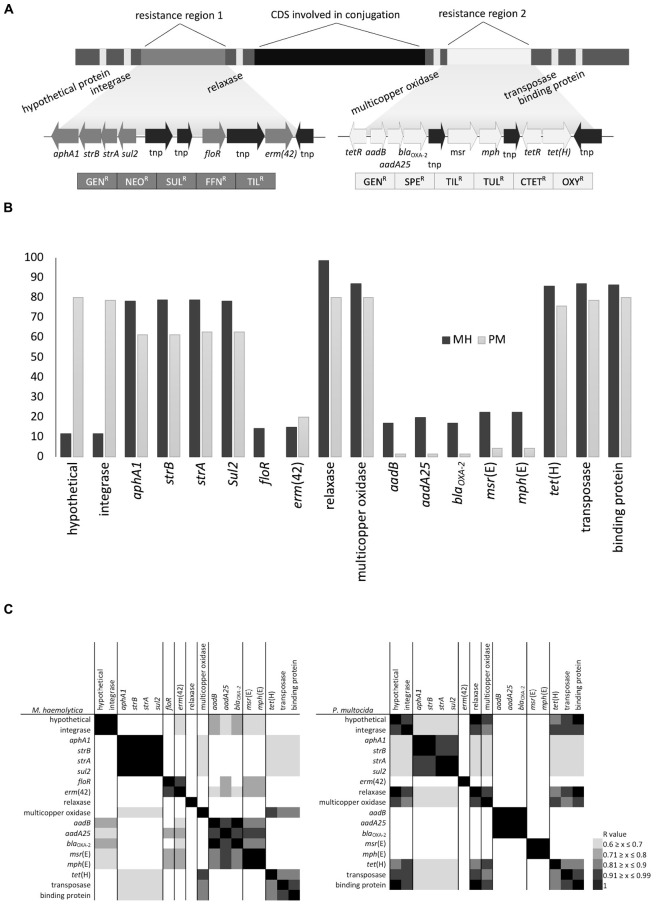
**(A)** Schematic representation of gene arrangement in ICE*Pmu1* originating from *Pasteurella multocida*, **(B)** percentage of isolates positive for antimicrobial resistance genes (ARGs) and ICE-associated genes, and **(C)** heatmaps displaying the association between ARGs and ICE-associated genes based on Pearson’s correlation coefficient.

As part of this study, PCR was used to screen for the presence of core and accessory genes identified in ICE*Pmu1.* At the time of the study design, ICE*Pmu*1 was one of the largest ICEs described in *Pasteurellaceae*, with the highest number of accessory genes, therefore its coding sequence was used to design all PCR primers and included five genes present within the functional gene regions of the ICE, and twelve ARGs and a multicopper oxidase gene present in two accessory gene cassettes known to be resistance regions (Figure 4A). The core genes targeted represented the full length of ICE*Pmu1* with the relaxase gene (Pmu_02890) from the central region, a hypothetical protein gene (Pmu_02680) and integrase gene (Pmu_02700) located on the left terminus and a transposase gene (Pmu_03510) and binding protein gene (Pmu_03540) located on the right terminus.

The majority (>80%) of *M. haemolytica* and *P. multocida* isolates harbored both the relaxase and multicopper oxidase genes located in the center of ICE*Pmu*1 along with the transposase and single-stranded DNA-binding protein genes on the right terminus. However, although 80% of *P. multocida* isolates carried the hypothetical and integrase genes present on the left flank of ICE*Pmu*1, less than 10% of *M. haemolytica* isolates were PCR positive for both of these genes indicating that differences may occur between species on the left flank of potential ICEs. The high prevalence of the multicopper oxidase gene in both species suggests that this gene is either closely linked with the functional backbone and not easily lost or is maintained due to selection pressure.

Although ICEs from *P. multocida* have been shown to transfer to *M. haemolytica in vitro* (Michael et al., 2012b) and strong similarities between ICEs from species within the *Pasteurellaceae* family have been previously reported (Beker et al., 2018; Bhatt et al., 2018; Klima et al., 2019), only two of the multidrug resistance profiles identified in this study were present in both species (Table 2). As well, the prevalence of individual ARGs was either high (60–80% of isolates) or low (0–20% of isolates) within the isolates screened (Figure 4B). There were, however, strong correlations between the presence of multiple ARGs in both species (Figure 4C) suggesting that regardless of abundance, some of these genes could be linearly arranged or linked in patterns that reflect gene arrangements within the resistance gene regions in ICE*Pmu1*. This is significant because linked genes typically transfer together and are more likely to be co-selected by the antimicrobials to which they confer resistance. In the case of *M. haemolytica*, *aphA1*, *strB*, *strA*, and *sul2*, *aadB* and *bla*_OXA–__2_, and *msr*(E) and *mph*(E), appeared to be linked based on Pearson’s correlation coefficient (*r* = 1, *P* ≤ 0.05). In *P. multocida*, linkages between *aphA1* and *strB, strA* and *sul2, aadB, aadA25*, *bla*_OXA–__2_, and *msr*(E) and *mph*(E) were also evident (*r* = 1, *P* ≤ 0.05). Similar ARG linkages to those seen here were also reported from the recent sequence analysis of over 1,000 MDR *M. haemolytica* genomes (Clawson et al., 2016).

Integrative and conjugative elements are thought to acquire accessory genes through the presence of insertion sequences, transposons, and specific recombinases within the ICE genome (Armshaw and Pembroke, 2013). Weaker positive correlations were observed (Pearson correlation *r* = 0.6 to 0.99, *P* ≤ 0.05) in this group of isolates that reflect ARGs adjacent to one another on ICE*Pmu1* and that are flanked by transposases [i.e., *aphaA1*/*strB*/*strA*/*sul2* and *floR* and *erm*(42), or *aadB*/*bla*_OXA–2_/*aadA25* and *msr*(E)/*mph*(E)]. It is likely that ARGs located next to transposases are part of insertion sequences and are independently mobile within these cassettes. If some genes are acquired or lost as groups while others are individually mobile, this could account for both the similarity in patterns and the overall diversity of multidrug resistance profiles observed in *Pasteurellaceae* ICEs. Previous work in our lab has shown that ARGs within ICE*MhL044A* (Klima et al., 2016) lacked post-conjugal stability, particularly *floR* and *erm*(42), which we have observed to be easily lost in recipient strains (C.L. Klima and T.A. McAllister, unpublished data). The instability of ARGs within existing ICEs would be positive from the standpoint of limiting AMR dissemination and spread. Many ICEs in *Pasteurellaceae* contain toxin-antitoxin systems that promote stability of the element within populations (Klima et al., 2016). However, if some ARGs in ICEs can be independently lost from their conserved functional regions, strategies to enhance ARG loss and not ICE loss could be developed to mitigate AMR in existing populations.

### Phenotype vs. Antimicrobial Resistance Genes Present in *M. haemolytica* and *P. multocida*

Although strong correlations were observed between AMR phenotype and ARGs, with the exception of florfenicol/*floR* in *M. haemolytica* isolates, none were completely correlated (Table 3). This may be due to other ARGs contributing to the phenotypes observed, the ARGs are inactive, or the breakpoints used for susceptibility testing need to be re-evaluated. The narrow-spectrum β–lactamase gene, *bla*_OXA–2,_ was the only ARG screened in association with β-lactam resistance (ampicillin, penicillin, ceftiofur). Previously reported in ICE*Pmu1*, *bla*_OXA–2_ is inactive in both host and recipient strains of *P. multocida* and *M. haemolytica* post-conjugation, but active when transferred into *Escherichia coli* (Michael et al., 2012b). Varied activity of *bla*_OXA–2_ based on bacterial host species has been observed before with the gene conferring extended-spectrum β-lactamase activity in *Acinetobacter baumannii*, an opportunistic nosocomial pathogen, but producing a narrow-spectrum antibiotic resistance pattern in *E. coli* (Antunes et al., 2014). Given the low concordance of *bla*_OXA–2_ with β-lactam resistance (<50%) in our study, its occurrence in 12.9% of susceptible *M. haemolytica* isolates suggests that it is inactive in these strains. Phenotypic resistance to β-lactams in *M. haemolytica* has been previously reported to be associated with *bla*_ROB–1_ (Klima et al., 2014b) and more recently with *bla*_ROB–2_ (Kadlec et al., 2018). It is possible that either of these genes could be contributing to the β-lactam resistance observed in the current study.

**TABLE 3:** Associations between antimicrobial resistance phenotype and antimicrobial resistance gene (ARG) *presence* in *Mannheimia haemolytica* (MH) and Pasteurella *multocida* (PM) isolates collected post-mortem from bovine respiratory disease mortalities.

Resistance phenotype/ARG	No. isolates with resistance phenotype MH| PM	% of the resistant isolates with ARG^a^ MH| PM	% of the susceptible isolates with ARG MH| PM
AMP/*bla*_OXA–2_	15 | 10	54 | 10	12.9 | 0
PEN/*bla*_OXA–2_	15 | 6	53.4 | 16.7	12.9 | 0
XNL/*bla*_OXA–2_	2 | 9	0 | 11.2	17.3 | 0
CTET/*tet*(H)	21 | 20	90.5 | 85	85 | 72
OXYT/*tet*(H)	131 | 61	96.2* | 86.9	0 | 0
FFN/*floR*	21 | 0	100** | 0	0 | 0
SDM/*sul2*	146 | 70	79.5 | 62.9	0 | 0
SXT/*sul2*	112 | 59	83.1 | 67.8	65.8 | 36.4
SPE/*aadA25*	32 | 44	90.7** | 2.3	0 | 0
SPE/*aadB*	32 | 44	78.2* | 2.3	0 | 0
NEO/*strA*	120 | 49	96.7** | 85.8*	0 | 9.6
NEO/*strB*	120 | 49	96.7** | 85.8*	0 | 4.8
NEO/*aphA1*	120 | 49	96.7** | 85.8*	0 | 4.8
GENT/*strA*	30 | 3	70 | 33.4	81.2 | 64.2
GENT/*strB*	30 | 3	70 | 33.4	81.2 | 62.7
GENT/ *aphA1*	30 | 3	70 | 33.4	81.2 | 62.7
GENT/*aadB*	30 | 3	83.4* | 33.4	0 | 0
GENT/*aadA25*	30 | 3	96.7** | 33.4	0 | 0
TUL/*msr*(E)	92 | 49	33.7 | 4.1	3.7 | 4.8
TUL/*mph*(E)	92 | 49	33.7 | 4.1	3.7 | 4.8
TUL *erm*(42)	92 | 49	22.9 | 8.2	1.9 | 47.7
TIL/*msr*(E)	97 | 62	29.9 | 4.9	8 | 0
TIL/*mph*(E)	97 | 62	29.9 | 4.9	8 | 0
TIL/*erm*(42)	97 | 62	22.7 | 22.6	0 | 0
TYLT/*msr*(E)	147 | 64	22.5 | 4.7	0 | 0
TYLT/*mph*(E)	147 | 64	22.5 | 4.7	0 | 0
TYLT/*erm*(42)	147 | 64	15 | 21.9	0 | 0
CLIND/*msr*(E)	57 | 70	42.2 | 4.3	10 | 0
CLIND/*mph*(E)	57 | 70	42.2 | 4.3	10 | 0
CLIND/*erm(*42)	57 | 70	38.6 | 20	0 | 0

^a^ Based on significant (P < 0.05) Pearson’s correlation coefficient; *r > 0.8, **r > 0.9.

The tetracycline efflux gene *tet*(H) was found in 90.5% of *M. haemolytica* and 85% of *P. multocida* tetracycline-resistant isolates. Interestingly, the association between *tet*(H) and tetracycline resistance (oxytetracycline, chlortetracycline) was significant in *M. haemolytica* expressing oxytetracycline resistance, but not chlortetracycline resistance. A large number (>70%) of *M. haemolytica* and *P. multocida* isolates were susceptible to chlortetracycline even though they carried *tet*(H), indicating that *tet*(H) does not always confer resistance to chlortetracycline. The prevalent resistance to oxytetracycline is not unexpected as it was administered as a therapeutic to animals surveyed here. Given that resistance to oxytetracycline was not 100% correlated with *tet*(H) it is possible that an alternative tetracycline resistance gene is conferring the phenotype observed. Although *tet*(H) has been the most commonly reported tetracycline resistance gene in North American *M. haemolytica* and *P. multocida* isolates, *tet*(M) and *tet*(B) have been identified in *P. multocida* from the United States (Hansen et al., 1996), *tet*(G) in *M. haemolytica* from Germany (Kehrenberg et al., 2001) and *P. multocida* from Taiwan (Wu et al., 2003), and *tet*(L) in *M. haemolytica* and *P. multocida* isolates from Belgium (Kehrenberg et al., 2005).

In terms of unresolved macrolide, sulfonamide, and sulfonamide/trimethoprim resistance, it is likely that ARGs other than those screened conferred resistance. Although *sul2* was present in many of the sulfadimethoxine- and trimethoprim/sulfamethoxazole-resistant isolates, it was absent in at least 20% of these isolates. We did not screen for the dihydrofolate reductase genes, *dfrA20* (Kehrenberg and Schwarz, 2005) or *dfrA14* (Niemann et al., 2019), that have been previously linked to trimethoprim resistance in *P. multocida.* Macrolide resistance was one of the most frequently observed phenotypes in both *M. haemolytica* and *P. multocida*, but the ARGs commonly affiliated with these phenotypes, [i.e., *erm*(42), *msr*(E), *mph*(E)], were not present in the majority of resistant isolates. Using functional libraries, we were unable to identify novel ARGs in strains here that lacked *erm*(42), *msr*(E), or *mph*(E) (C.L. Klima and T.A. McAllister, unpublished data), suggesting that macrolide resistance is likely associated with mutations in the 23S rRNA gene that can confer high-level resistance to macrolides in both *M. haemolytica* and *P. multocida* (Olsen et al., 2015). There were many incidences of clindamycin resistance where *erm*(42) was not present, likely indicating that an alternative mechanism is contributing to clindamycin resistance.

Although *aadB* and *aadA25* are both commonly present in spectinomycin-resistant *M. haemolytica*, *aadA25* was detected in 90.7% of these isolates and was more strongly associated with the spectinomycin-resistant phenotype than *aadB*. Neither gene was highly prevalent (<3%) in the 62.8% of *P. multocida* that were spectinomycin-resistant, but mutations in both the 16S rRNA and *rpsE* genes have been reported to confer high-level resistance to spectinomycin in *P. multocida* (Kehrenberg and Schwarz, 2007). Incidentally, *aadA25* was strongly associated with gentamicin resistance in *M. haemolytica*, and identified in 96.7% of gentamicin-resistant isolates. Although the *strA*, *strB*, and *aphA1* genes were all present in *M. haemolytica* and *P. multocida* neomycin^–^and gentamicin-resistant isolates, they were more often (>85%) associated with neomycin resistance.

Despite the fact that multiple ARGs that typically confer resistance to aminoglycosides were detected, no single gene was found to be 100% associated with any of the resistance phenotypes. As a result, it is probable that another aminoglycoside resistance gene is also contributing to the resistance profiles seen; possibly the newly described *aadA31* (Cameron et al., 2018) or *aadA15* (Clawson et al., 2016) genes that have been recently reported in *M. haemolytica*.

### Relationships Between Multidrug Resistance Profiles and Metadata Categories

**FIGURE 5:**
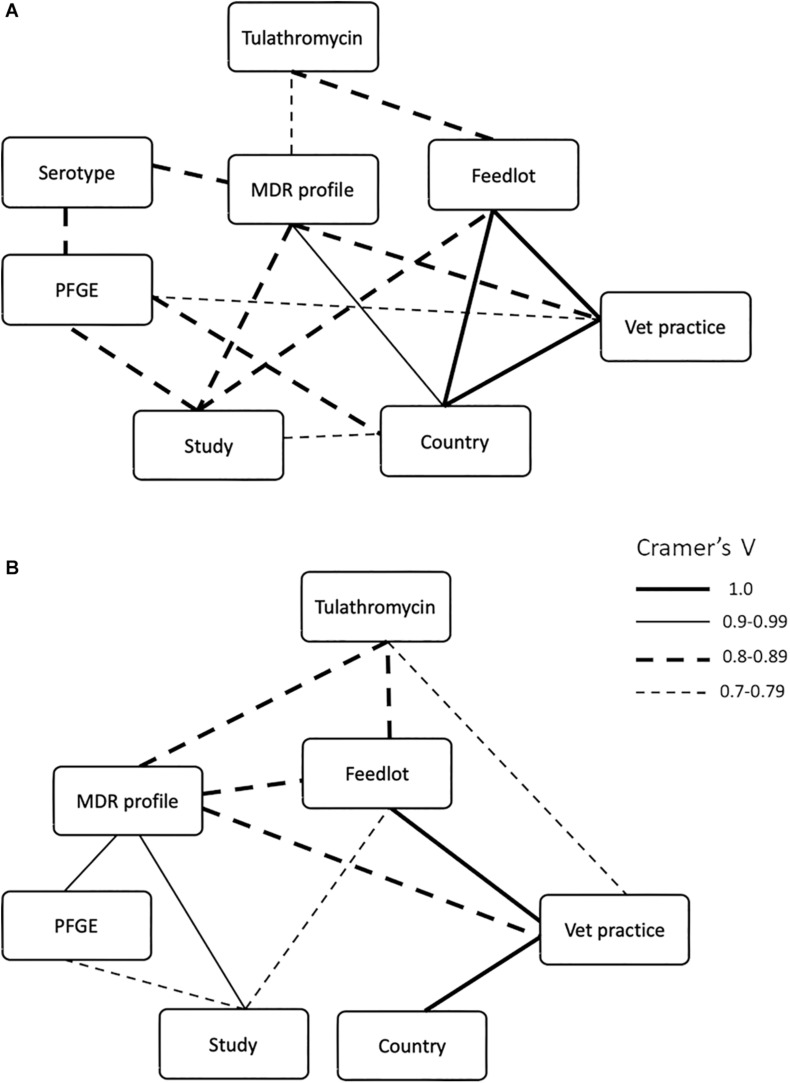
Significant (Cramer’s *V* ≥ 0.7, *P* ≤ 0.05) associations between multidrug resistance profiles of **(A)** *Mannheimia haemolytica* and **(B)** *Pasteurella multocida* isolates with feedlot, country of origin, submitting veterinary clinic, diagnosis, PFGE profile, serotype (for *M. haemolytica*), and treatment. Weight of the lines correspond to Cramer’s *V* values.

Metadata, including feedlot, country, submitting veterinary clinic, diagnosis, multidrug resistance profile, PFGE profile, serotype (for *M. haemolytica*), and treatment, were collected for samples from all three studies and the strength of association among these nominal (categorical) data was determined using Cramer’s *V* (Figure 5). Because these isolates were collected from naturally occurring mortality cases and are specifically the MDR subset (representing anywhere from 0 to 100% of isolates collected depending on location), there is a skew in the number of strains recovered from the different feedlots examined (Supplementary Table S1). Consequently, the statistical analyses used were designed to examine data of this type, specifically non-parametric nominal data. As might be expected of bacterial populations existing in closed environments, strong associations were observed in both bacterial species between multidrug resistance profile and any factor associated with the origin of the isolates, including year, country of isolate origin, feedlot, or the veterinary practice that collected the sample. For *M. haemolytica*, the multidrug resistance profile was strongly associated with study (Cramer’s *V* = 0.841, *P* = 0.0004), country of origin (Cramer’s *V* = 0.961, *P* = 0.0004), veterinary practice (Cramer’s *V* = 0.850, *P* = 0.0004), serotype (Cramer’s *V* = 0.845, *P* = 0.0166), and the use of tulathromycin (Cramer’s *V* = 0.796, *P* = 0.0004) (Figure 5A). For *P. multocida*, the multidrug resistance profile was strongly associated with the study (Cramer’s *V* = 0.915, *P* = 0.0007), feedlot (Cramer’s *V* = 0.891, *P* = 0.0007), veterinary practice (Cramer’s *V* = 0.887, *P* = 0.0007), PFGE profile (Cramer’s *V* = 0.917, *P* = 0.0007) and the use of tulathromycin (Cramer’s *V* = 0.803, *P* = 0.0092) (Figure 5B). Although not exclusive, most *M*. *haemolytica* isolates were collected from cattle with a diagnosis of fibrinous pneumonia (67%), while *P*. *multocida* isolates were most often collected from bronchopneumonia (40%) cases (Supplementary Table S4).

**FIGURE 6:**
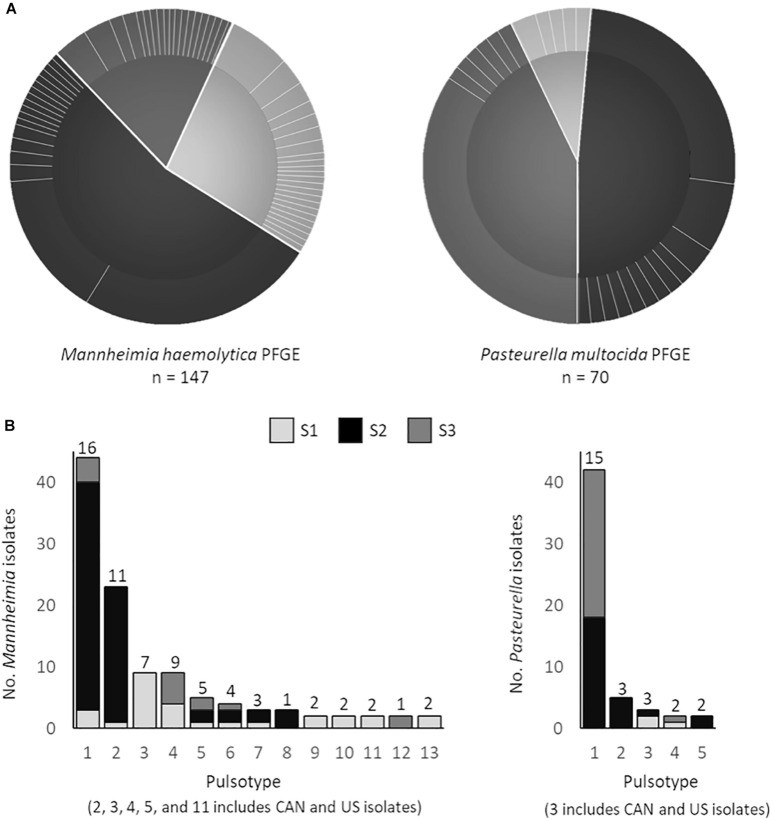
**(A)** Pulsed-field gel electrophoresis (PFGE) profiles of *Mannheimia haemolytica* (SalI) and *Pasteurella multocida* (ApaI) isolates. Pie charts depict the number of unique pulsotypes (>95% similarity) per study (study 1 *M*. *haemolytica* = 40, *P*. *multocida* = 6; study 2 *M*. *haemolytica* = 80, *P*. *multocida* = 34; study 3 *M*. *haemolytica* = 27, *P*. *multocida* = 34) and the proportion of isolates with each pulsotype. **(B)** PFGE profiles that contain strains from multiple locations. Numbers above each bar represent the number of unique antimicrobial susceptibility profiles within each unique PFGE profile.

The PFGE data, which reflects overall genetic content rather than only multidrug resistance profile highlight less geographic-based differences than the correlation statistics indicate. Of the 21 unique pulsotypes identified in *P. multocida*, a single pulsotype was observed in 60% of all isolates originating from 13 feedlots. Furthermore, of the 53 unique pulsotypes in *M. haemolytica*, two pulsotypes accounted for 45% of all *M. haemolytica* isolates, from 13 to 7 feedlots, respectively (Figure 6A). Five of the thirteen *M. haemolytica* pulsotypes found in multiple locations were detected in both the United States and Canada (Figure 6B). None of the *P. multocida* pulsotypes were shared between the United States and Canada, however, the total number of isolates examined was half that of *M. haemolytica.* The three *M. haemolytica* pulsotypes found in all three studies and the three pulsotypes of *P. multocida* that span two studies, indicate that MDR *Pasteurellaceae* are widely disseminated within the cattle populations investigated. However, whole genome sequence analysis will be required to determine if the same strains are present across these times and distances.

Of note, there was no association between pulsotype (PFGE) and multidrug resistance profile (Figure 5), indicating that isolates with similar genotypes may contain MGEs that carry different ARGs. As a result, genotype based on PFGE is likely not a good metric to track the spread of AMR associated with MGEs. Whole genome sequencing is becoming standard practice for surveillance studies, but where funds or infrastructure for genomics analysis is limited, diagnostics that trace the movement of unique MGEs may be more helpful in assessing AMR spread compared to strain typing.

### Multidrug Resistance and *M*. *haemolytica* Serotype

An association was found between multidrug resistance profile and serotype in the *M. haemolytica* isolates (Cramer’s *V* = 0.8453, *P* = 0.0082; Figure 5A), with 91.8% of the isolates being serotype 1 (Supplementary Table S5). Although the correlation between serovar and PFGE profile indicates that serovar is linked with genotype (Figure 5A), an overlap in the multidrug resistance profile between serotype 1 and serotype 6 isolates (five out of 45 multidrug resistance profiles) suggests that MGEs likely transfer between serovars and that genotype is not a strong correlate of AMR profile.

Of the three serovars of *M. haemolytica* commonly found in cattle, serotype 2 is considered less virulent and is often isolated from the nasopharynx of healthy cattle (Klima et al., 2014a). Although not typically targeted by antimicrobial therapy, serotype 2 strains were resistant to six and 10 drugs, respectively. This suggests that serotype 2 strains could act as a reservoir of ARGs in healthy cattle populations even though they are not directly implicated in BRD. As serotype 2 strains are considered to be more prevalent in healthy cattle, they may make a larger contribution to the respiratory tract resistome than do serotypes 1 and 6.

### Antimicrobial Resistance in *M*. *haemolytica* and *P. multocida* Associated With Antimicrobial Use

Of the veterinary clinics that submitted samples, all but one was willing to provide therapeutic drug administration data. The remaining clinic was willing to provide how many times an animal was treated but not the specific drugs or dosages used. No information was available from any of the feedlots regarding the antimicrobials that were administered infeed. However, it is reasonable to assume, given standard industry practices at the time of these studies, that the majority of these animals had either tylosin and/or chlortetracycline included in their diets. Although previous work has shown a relationship between the frequency of antimicrobial exposure and the amount of AMR observed in *M. haemolytica* and *P. multocida* (Magstadt et al., 2018), no strong associations were observed between multidrug resistance and the number of treatments each animal received (*M. haemolytica*; Cramer’s *V* = 0.396, *P* = 0.048; *P. multocida*; Cramer’s *V* = 0.3307, *P* > 0.05). In fact, there were multiple cases where isolates resistant to four or five antimicrobials were recovered from animals treated up to four times, whereas isolates resistant to 12 to 15 antimicrobials were recovered from cattle that received only one to three antimicrobial treatments (Table 4). There was also no strong association between the individual antimicrobial used and the year of sample collection (Cramer’s *V* < 0.5).

**TABLE 4:** Percentage (No.) of cattle that had been treated multiple times as a result of bovine respiratory disease (BRD).

	Percentage of BRD cases by number of antimicrobials treated (No. animals)						
Bacterial species isolated	None	Once	Twice	Three	Four	Five	Six
*Mannheimia haemolytica*	33 (48)	39 (58)	17 (25)	7 (11)	3 (4)	1 (1)	–
*Pasteurella multocida*	34 (24)	21 (15)	11 (8)	7 (5)	13 (9)	10 (7)	3 (2)

**FIGURE 7:**
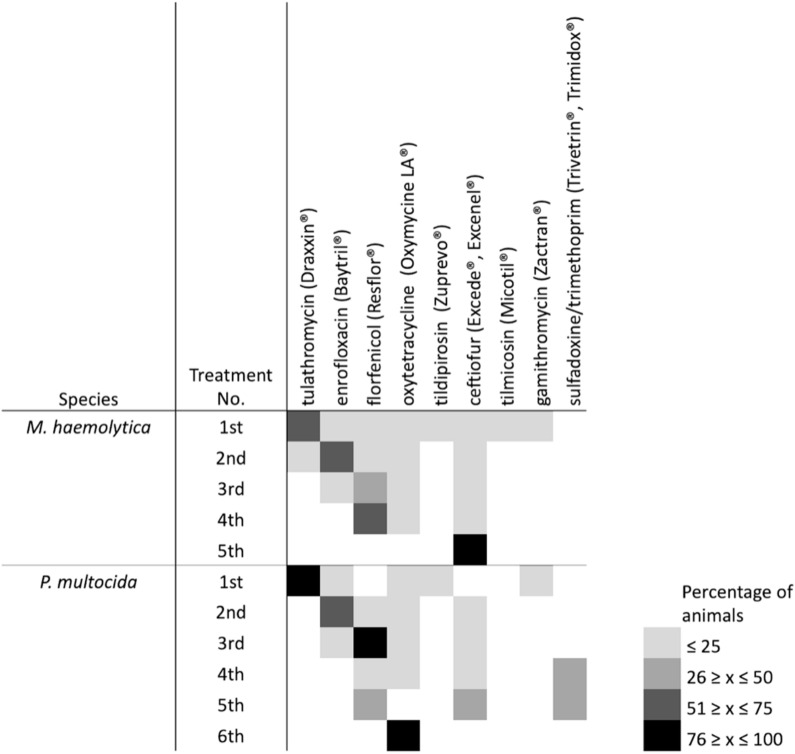
Heatmap of the antimicrobial treatment data for animals examined post-mortem after having died of bovine respiratory disease. Box color indicates overall percentage of animals that received an antimicrobial based on the data that was provided by veterinary clinics.

Over 65% of mortalities sampled were from cattle that had received at least one antimicrobial treatment, with the majority being treated only once (Table 5). Tulathromycin, enrofloxacin, florfenicol, ceftiofur and oxytetracycline were the antimicrobials most commonly administered (Figure 7), with tulathromycin most often given as a first-line treatment, enrofloxacin second, and florfenicol third. Of the cattle that were treated multiple times, ceftiofur, florfenicol, or oxytetracycline were most frequently used. The aforementioned antimicrobials were most commonly administered to cattle from which *M. haemolytica* was isolated, while a combination of trimethoprim-sulfadoxine or oxytetracycline were most often administered to cattle from which P. *multocida* was isolated. No associations were found between gross pathology (i.e., bronchointerstitial pneumonia vs. fibrinous pleuritis) and the antimicrobials administered, suggesting that any form of respiratory illness, regardless of its presentation, is likely managed similarly, among different feedlots.

**TABLE 5:** Percentage (No.) of *Mannheimia haemolytica* and *Pasteurella multocida* isolates from bovine respiratory disease mortalities that are multidrug resistant by number of antimicrobial treatments the animal received.

		Percentage of isolates (no. isolates) with drug count of multidrug resistance profile												
Species	Treatment No.	3	4	5	6	7	8	9	10	11	12	13	15	16
*M. haemolytica*	None	4 (2)	15 (7)	19 (9)	15 (7)	17 (8)	2 (1)	2 (1)	8 (4)	13 (6)	–	–	–	6 (3)
	First	3 (2)	7 (4)	9 (5)	5 (3)	52 (30)	10 (6)	2 (1)	5 (3)	3 (2)	–	2 (1)	2 (1)	–
	Second	8 (2)	12 (3)	8 (2)	–	36 (9)	12 (3)	12 (3)	4 (1)	4 (1)	–	–	4 (1)	–
	Third	–	18 (2)	–	–	18 (2)	9 (1)	9 (1)	–	–	9 (1)	9 (1)	27 (3)	–
	Fourth	–	25 (1)	–	50 (2)	25 (1)	–	–	–	–	–	–	–	–
	Fifth	100 (1)	–	–	–	–	–	–	–	–	–	–	–	–
*P. multocida*	None	–	4 (1)	4 (1)	4 (1)	13 (3)	21 (5)	25 (6)	17 (4)	8 (2)	4 (1)	–		
	First	–	7 (1)	7 (1)	–	–	7 (1)	27 (4)	33 (5)	7 (1)	13 (2)	–		
	Second	25 (2)	–	13 (1)	–	–	–	13 (1)	38 (3)	–	–	13 (1)		
	Third	–	–	–	–	–	–	20 (1)	40 (2)	–	–	40 (2)		
	Fourth	11 (1)	–	–	–	–	–	11 (1)	56 (5)	22 (2)	–	–		
	Fifth	–	–	–	–	–	–	43 (3)	43 (3)	14 (1)	–	–		
	Sixth	–	–	–	–	–	–	–	50 (1)	50 (1)	–	–		

For MDR *M. haemolytica* isolates, strong associations were observed between multidrug resistance profile and usage of ceftiofur (Cramer’s *V* = 0.7172, *P* = 0.0423) and tulathromycin (Cramer’s *V* = 0.7019, *P* = 0.0004), with weaker associations found between multidrug resistance profile and the use of florfenicol (Cramer’s *V* = 0.6765, *P* = 0.0288). Strong associations were only observed in MDR *P*. *multocida* with tulathromycin use (Cramer’s *V* = 0.8025, *P* = 0.0129).

### Perspectives on Antimicrobial Resistance in Beef Cattle

A key function of AMR surveillance is to assess risks associated with antimicrobial use in food production settings. However, beef production systems vary widely. Feedlots can range significantly in size (3,000 to 100,000 head), how animals are procured (auction vs. direct sourced), the risk category of animals that operators are willing to purchase, the distance cattle are shipped, drug regimens used to prevent and treat disease, and how animals are commingled and moved within the feedlot environment. As a result, the information obtained here cannot effectively address how antimicrobial use may be affecting AMR development and spread in all types of beef production.

What the data do support is that possible XDR *M. haemolytica* and *P. multocida* are prevalent in mortality cases associated with BRD in North America. Despite not being frequently observed in BRD cases prior to Lubbers and Hanzlicek (2013), the AMR profiles are both diverse and persistent. The increased multidrug resistance observed during the 7 years of study, in combination with the abundance of ICE-associated genes present in *M*. *haemolytica* and *P*. *multocida*, suggests that ICE carrying ARGs are likely contributing to treatment failures in cases of respiratory infection in North American cattle. The linkage between the use of tulathromycin, MDR, and ICEs likely reflects the extensive metaphylactic use of this antimicrobial in Canadian feedlots (Brault et al., 2019) Further examination is warranted to determine if ICEs are arising independently through recombination with plasmids and other MGEs, or if the majority of the AMR observed is due to a common element that is transferred and then modified through gene loss or uptake dependent upon selective pressures. The selective pressures promoting ICE spread are also not known. The diversity in both AMR phenotype and the ARGs detected indicate that there is likely more genetic diversity than what has been previously described in ICE from *Pasteurellaceae* spp.

## Conclusion

This study suggests that between 2011 and 2016 there was a significant increase in the prevalence of MDR isolates of *M. haemolytica* and *P. multocida* associated with BRD. Furthermore, the AMR profiles observed were diverse and persistent over time. Given the presence of multiple genes associated with both functional gene coding regions and accessory gene cassettes previously reported in ICEs from *Pasteurellaceae*, it is likely that similar ICEs are abundant in the populations examined. The complexity of the multidrug resistance profiles and the lack of correlation with genotype suggests that horizontal gene transfer is contributing to the spread of multidrug resistance in *Pasteurellaceae* spp. in cattle. Interestingly, the occurrence of resistance to more than seven antimicrobials in *M. haemolytica* decreased over time but increased in *P. multocida* indicating that the dynamics of multidrug resistance spread may differ amongst species that contribute to BRD. There was no correlation with number of antimicrobial treatments individual animals received and the number or types of drugs to which isolates were resistant. A multicopper oxidase gene was prevalent among the isolates and may be a target of selection pressure and play a role in ICE maintenance and spread in feedlots. To address concerns over multidrug resistance spread in beef production in North America, more information regarding the diversity, prevalence, and host range of ICEs in *Pasteurellaceae* is required. To gain a greater understanding of ICEs within these populations, including how they are developing and spreading will likely require combined efforts amongst industry, veterinarians, producers, and researchers.

## Ethics Statement

The animal study was reviewed and approved by Lethbridge Research Centre Animal Care Committee. Written informed consent was obtained from the owners for the participation of their animals in this study.

## Author Contributions

CK and TM designed the experiment BR, CB, SH, NA, RM, KS, and TM provided the study samples and metadata. SC, CC, and YN supported the technical aspects of the study and carried out experiments. CK carried out the experiments and wrote the manuscript. CK and DH analyzed the data. All authors revised and approved the manuscript to be published.

## Conflict of Interest

CB is part owner and managing partner of Feedlot Health Management Services Ltd. and Southern Alberta Veterinary Services Ltd. SH is an employee of Feedlot Health Management Services Ltd. RM is owner of POV Inc. The remaining authors declare that the research was conducted in the absence of any commercial or financial relationships that could be construed as a potential conflict of interest.
