# Corrigendum: Multidrug Resistance in *Pasteurellaceae* Associated With Bovine Respiratory Disease Mortalities in North America From 2011 to 2016

**DOI:** 10.3389/fmicb.2020.638008

**Published:** 2021-02-02

**Authors:** Cassidy L. Klima, Devin B. Holman, Shaun R. Cook, Cheyenne C. Conrad, Brenda J. Ralston, Nick Allan, R. Michele Anholt, Yan D. Niu, Kim Stanford, Sherry J. Hannon, Calvin W. Booker, Tim A. McAllister

**Affiliations:** ^1^Lethbridge Research and Development Centre, Agriculture and Agri-Food Canada, Lethbridge, AB, Canada; ^2^Lacombe Research and Development Centre, Agriculture and Agri-Food Canada, Lacombe, AB, Canada; ^3^Alberta Agriculture and Forestry, Lethbridge, AB, Canada; ^4^Alberta Agriculture and Forestry, Airdrie, AB, Canada; ^5^Chinook Contract Research Inc., Airdrie, AB, Canada; ^6^POV Inc., Airdrie, AB, Canada; ^7^Faculty of Veterinary Medicine, University of Calgary, Calgary, AB, Canada; ^8^Department of Biological Sciences, University of Lethbridge, Lethbridge, AB, Canada; ^9^Feedlot Health Management Services Ltd, Okotoks, AB, Canada

**Keywords:** antimicrobial resistance, bovine respiratory disease, *Mannheimia haemolytica*, *Pasteurella multocida*, integrative and conjugative elements, *Pasteurellaceae*

In the original article, an incorrect Copyright statement was published. The corrected Copyright statement should read:

Copyright ©2020 Klima, Holman, Cook, Conrad, Ralston, Allan, Anholt, Niu, Stanford, Hannon, Booker, McAllister and Her Majesty the Queen in Right of Canada, as represented by the Minister of Agriculture and Agri-Food Canada. This is an open-access article distributed under the terms of the Creative Commons Attribution License (CC BY). The use, distribution or reproduction in other forums is permitted, provided the original author(s) and the copyright owner(s) are credited and that the original publication in this journal is cited, in accordance with accepted academic practice. No use, distribution or reproduction is permitted which does not comply with these terms.

The authors apologize for this error and state that this does not change the scientific conclusions of the article in any way. The original article has been updated.

