# Supplementary material for: Multidrug Resistance in Pasteurellaceae Associated With Bovine Respiratory Disease Mortalities in North America From 2011 to 2016
Source: Front Microbiol. 2020 Nov 5;11:606438. doi: 10.3389/fmicb.2020.606438 (PMC7682020; doi:10.3389/fmicb.2020.606438)
Supplement: Supplementary file 1 [file Data_Sheet_1.docx]

**Supplementary Table S1.** Number of multidrug-resistant isolates collected from each feedlot during each study.

| **Feedlot ID** | **Study 1** | **Study 2** | **Study 3** | **Total** |
| --- | --- | --- | --- | --- |
| ***Mannheimia haemolytica*** |  |  |  |  |
| 1 | 1 | . | . | 1 |
| 2 | 3 | . | . | 3 |
| 3 | 2 | . | . | 2 |
| 4 | 2 | . | . | 2 |
| 5 | 1 | . | . | 1 |
| 6 | 2 | . | . | 2 |
| 7 | 1 | . | . | 1 |
| 8 | 3 | . | . | 3 |
| 9 | . | 1 | . | 1 |
| 10 | 1 | . | . | 1 |
| 11 | . | . | 1 | 1 |
| 12 | . | . | 1 | 1 |
| 13 | 5 | . | . | 5 |
| 14 | 19 | . | . | 19 |
| 15 | . | 1 | . | 1 |
| 16 | . | . | 2 | 2 |
| 17 | . | 3 | 1 | 4 |
| 18 | . | 2 | 2 | 4 |
| 19 | . | 1 | . | 1 |
| 20 | . | 4 | . | 4 |
| 21 | . | 0 | 4 | 4 |
| 22 | . | 1 | 0 | 1 |
| 23 | . | 1 | . | 1 |
| 24 | . | 1 | . | 1 |
| 25 | . | 1 | 1 | 2 |
| 26 | . | 1 | . | 1 |
| 27 | . | 47 | 14 | 61 |
| 28 | . | 3 | 1 | 4 |
| 29 | . | 2 | . | 2 |
| 30 | . | 1 | . | 1 |
| 31 | . | 2 | . | 2 |
| 32 | . | 8 | 0 | 8 |
| ***Pasteurella multocida*** |  |  |  |  |
| 2 | 1 | . | . | 1 |
| 5 | 1 | . | . | 1 |
| 6 | 1 | . | . | 1 |
| 8 | 1 | . | . | 1 |
| 12 | . | . | 1 | 1 |
| 13 | 1 | . | . | 1 |
| 14 | 1 | . | . | 1 |
| 16 | . | . | 1 | 1 |
| 17 | . | 1 | 1 | 2 |
| 18 | . | 0 | 1 | 1 |
| 20 | . | 1 | . | 1 |
| 21 | 1 | . | 2 | 3 |
| 22 | . | 0 | 1 | 1 |
| 24 | . | 1 | . | 1 |
| 25 | . | 22 | 14 | 36 |
| 26 | . | 4 | 4 | 8 |
| 28 | . | 2 | 2 | 4 |
| 32 | . | 2 | 1 | 3 |
| 33 | . | . | 2 | 2 |

^a^. Indicates that samples were not collected from this feedlot during the corresponding study.

| **Supplementary Table S2**. Minimum inhibitory concentrations (MIC) for each antimicrobial for *Mannheimia haemolytica* and *Pasteurella* *multocida* isolates collected post-mortem from bovine respiratory disease mortalities in North America. | | | | | | | | | | | | | | |  |
| --- | --- | --- | --- | --- | --- | --- | --- | --- | --- | --- | --- | --- | --- | --- | --- |
|  |  |  | **Distribution (%) of MICs in µg/mL (study1/study2/study3)** | | | | | | | | | |  |  |  |
| **Class** | **Antimicrobial ^(^Category^a^)^b^** | **Species** | **0.12** | **0.25** | **0.5** | **1** | **2** | **4** | **8** | **16** | **32** | **64** | **≥2/38** | **≥256** | **Isolates (%) resistant (Study1/Study2/Study3)** |
| Aminoglycosides | GEN (II)^2^ | *M. hameolytica* |  |  |  | 38/1/74 | 8/18/15 | 0/73/0 | 0/3/0 | **55/6/11** |  |  |  |  | 55/6/11 |
|  |  | *P. multocida* |  |  |  | 0/3/10 | 67/6/53 | 17/41/37 | 0/44/0 | **17/6/0** |  |  |  |  | 17/6/0 |
|  | NEO (II)^2^ | *M. hameolytica* |  |  |  |  |  | 30/0/41 | 3/3/0 | 0/1/0 | **68/96/59** |  |  |  | 68/96/59 |
|  |  | *P. multocida* |  |  |  |  |  | 0/0/13 | 50/3/27 | 0/12/3 | **50/85/57** |  |  |  | 50/85/57 |
|  | SPE (III) ^2^ | *M. hameolytica* |  |  |  |  |  |  | 0/1/0 | 45/6/78 | 0/85/7 | **55/8/15** |  |  | 55/8/15 |
|  |  | *P. multocida* |  |  |  |  |  |  | 0/3/3 | 67/29/10 | 17/18/0 | **17/50/87** |  |  | 17/50/87 |
|  | ENRO (I)^1^ | *M. hameolytica* | 45/94/89 | 20/1/0 | 20/0/0 | 0/0/0 | **15/5/11** |  |  |  |  |  |  |  | 15/5/11 |
|  |  | *P. multocida* | 83/88/83 | 0/9/17 | 17/3/0 | 0/0/0 | **0/0/0** |  |  |  |  |  |  |  | 0/0/0 |
|  | DANO (I) ^1^ | *M. hameolytica* | 45/93/89 | 3/3/0 | 38/0/0 | **15/5/11** |  |  |  |  |  |  |  |  | 15/5/11 |
|  |  | *P. multocida* | 67/94/70 | 0/6/7 | 17/0/17 | **17/0/7** |  |  |  |  |  |  |  |  | 17/0./7 |
| Macrolides | TYLT (II) ^2^ | *M. hameolytica* |  |  | 0/0/0 | 0/0/0 | 0/0/0 | 0/0/0 | 0/0/0 | 0/0/0 | **100/100/100** |  |  |  | 100/100/100 |
|  |  | *P. multocida* |  |  | 0/0/3 | 0/0/0 | 0/0/0 | 0/0/0 | 17/0/0 | 17/6/3 | **67/94/93** |  |  |  | 67/94/93 |
|  | TUL (II) ^1^ | *M. hameolytica* |  |  |  | 0/0/0 | 5/0/22 | 8/1/19 | 8/5/7 | 20/16/0 | 3/8/4 | **58/70/48** |  |  | 58/70/48 |
|  |  | *P. multocida* |  |  |  | 50/3/3 | 0/3/0 | 33/3/0 | 0/3/3 | 0/24/0 | 17/3/0 | **0/62/93** |  |  | 0/62/93 |
|  | TIL (II) ^1^ | *M. hameolytica* |  |  |  |  |  | 13/3/41 | 25/3/7 | 8/19/0 | **18/6/4** | **38/70/48** |  |  | 55/76/52 |
|  |  | *P. multocida* |  |  |  |  |  | 33/3/3 | 17/3/3 | 0/3/0 | **33/3/0.** | **17/88/93** |  |  | 50/91/93 |
| ß-lactams | XNL (I) ^1^ | *M. hameolytica* |  | 98/98/81 | 3/0/11 | 0/1/4 | 0/0/0 | 0/0/0 | **0/1/4** |  |  |  |  |  | 13/8/15 |
|  |  | *P. multocida* |  | 0/97/7 | 0/0/10 | 67/0/23 | 0/0/23 | 0/0/10 | **33/3/27** |  |  |  |  |  | 33/3/27 |
|  | PEN (II) ^2^ | *M. hameolytica* | 43/50/67 | 25/40/19 | 13/3/0 | 8/0/0 | 0/0/0 | 0/0/0 | **13/8/15** |  |  |  |  |  | 13/8/15 |
|  |  | *P. multocida* | 0/12/13 | 0/65/3 | 17/24/17 | 50/0/30 | 0/0/20 | **17/0/3** | **17/0/13** |  |  |  |  |  | 33/0/17 |
|  | AMP (II) ^2^ | *M. hameolytica* |  | 88/90/85 | 0/3/0 | 0/0/0 | 0/0/0 | **0/0/4** | **0/0/0** | **13/8/11** |  |  |  |  | 13/8/15 |
|  |  | *P. multocida* |  | 0/62/10 | 0/38/3 | 17/0/20 | 50/0/40 | **0/0/10** | **0/0/3** | **33/0/13** |  |  |  |  | 33/0/27 |
| Lincosamides | CLIN (II) ^2^ | *M. hameolytica* |  | 0/0/7 | 0/0/0 | 0/0/0 | 0/0/0 | 8/26/0 | 40/54/19 | **53/20/74** |  |  |  |  | 53/20/74 |
|  |  | *P. multocida* |  | 0/0/0 | 0/0/0 | 0/0/0 | 0/0/0 | 0/0/0 | 0/0/0 | **100/100/100** |  |  |  |  | 100/100/100 |
| Phenicol | FFN (III) ^1^ | *M. hameolytica* |  | 0/0/0 | 53/14/78 | 13/78/4 | 0/1/7 | 3/1/0 | **33/6/11** |  |  |  |  |  | 33/6/11 |
|  |  | *P. multocida* |  | 50/0/10 | 50/41/77 | 0/56/13 | 0/3/0 | 0/0/0 | **0/0/0** |  |  |  |  |  | 0/0/0 |
| Tetracycline | OXY (III) ^2^ | *M. hameolytica* |  |  | 0/0/41 | 10/1/0 | **0/1/0** | **0/1/0** | **90/96/59** |  |  |  |  |  | 90/99/59 |
|  |  | *P. multocida* |  |  | 0/0/3 | 33/3/3 | 17/0/0 | 0/9/0 | **50/88/93** |  |  |  |  |  | 50/88/93 |
|  | CTET (III) ^1^ | *M. hameolytica* |  |  | 0/0/41 | 10/4/7 | 30/40/41 | 38/43/7 | **23/14/4** |  |  |  |  |  | 23/14/4 |
|  |  | *P. multocida* |  |  | 33/0/10 | 17/6/83 | 0/18/3 | 33/24/0 | **17/53/3** |  |  |  |  |  | 17/53/3 |
| Pleuromutilin | TIA (III) ^2^ | *M. hameolytica* |  |  | 0/0/0 | 0/0/0 | 0/1/0 | 0/0/0 | 53/9/41 | 48/79/59 | **0/11/0** |  |  |  | 0/11/0 |
|  |  | *P. multocida* |  |  | 0/0/0 | 0/0/0 | 0/0/0 | 0/0/0 | 33/0/0 | 33/6/33 | **33/94/67** |  |  |  | 33/94/67 |
| Sulfonamide | SXT (II) ^2^ | *M. hameolytica* |  |  |  |  |  |  |  |  |  |  | 30/80/74 |  | 30/80/74 |
|  |  | *P. multocida* |  |  |  |  |  |  |  |  |  |  | 0/100/83 |  | 0/100/83 |
|  | SDM (III) ^2^ | *M. hameolytica* |  |  |  |  |  |  |  |  |  |  |  | 98/100/100 | 98/100/100 |
|  |  | *P. multocida* |  |  |  |  |  |  |  |  |  |  |  | 100/100/100 | 100/100/100 |
|  |  |  |  |  |  |  |  |  |  |  |  |  |  |  |  |

Trimethoprim sulfamethoxazole^c^ (Category II): <2/38 = 98.3%, >2/38 = 1.7%.

Sulfahdimethoxine^c^ (Category III): <256 = 44.6%, >256 = 55.4%.

GEN, gentamicin/NEO, neomycin/SPE, spectinomycin; DAN, danofloxacin/ENRO, enrofloxacin; TYLT, tylosin/TUL, tulathromycin/TIL, tilmicosin; PEN, penicillin/AMP, ampicillin/XNL, ceftiofur; CLI, clindamycin; FFN, florfenicol; SXT, trimethoprim sulfamethoxazole/SDM, sulfadimethoxine; OXY, oxytetracycline/CTET, chlortetracycline; TIA, tiamulin.

^a^Categorization of antimicrobial drugs based on importance in human medicine—Canadian Veterinary Drug Directorate.

^b^Breakpoints derived from CLSI guidelines (2..8)^1^ or MICs that correlated with presence of ARG^2^ and are indicated in bold.

Shaded areas indicate concentrations not tested.

**Supplementary Table S3**. PCR primers, amplicon size and primer concentration used for antimicrobial resistance gene screening.

| Assay name | Target | Forward (5' to 3') | Reverse (5' to 3') | Amplicon size (bp) | Primer concentration (nM) |
| --- | --- | --- | --- | --- | --- |
| RR1-MPLEX | *floR* | CGGCCTTTGTCGCTTTCCGTCT | CCGCAAACAAGCAACGCCATCC | 565 | 100 |
|  | *strB* | GCGGAACTGATGGCGAAGCTGT | TGCGGAAAGGCACCCATAAGCG | 441 | 100 |
|  | *aphA1* | GTCGGGCAATCAGGTGCGACAA | GATTGCGCCTGAGCGAGACGAA | 365 | 100 |
|  | *strA* | CGCAATGCCGTCAATCCCGACT | AAGGCGCGCTCTGCTTCATCTG | 299 | 100 |
|  | *sul2* | CCGGTATCAAACGCAACCGCCT | GAAGCGCAGCCGCAATTCATCG | 116 | 100 |
|  |  |  |  |  |  |
| RR2-MPLEX | MCO | ACGGACAACTACCAGGGCCACA | GCAGTTCATCAACAGGCGAGCG | 591 | 100 |
|  | *tet*(H) | AGGCGCGGCACTCGACTATCTT | AGCACCCAGATGGTGGCAGGAA | 465 | 100 |
|  | *bla*_OXA-2_ | AGGGGCTTTGCAGGCCACAATC | CAGCCCGTCTTTGCACGCAGTA | 335 | 100 |
|  | *aadA25* | GCGTCATTGAGCGCCATCTGGA | TAGCCGGATAACGCCACGGGAT | 258 | 100 |
|  | *aadB* | ATGCACGGCTAGGGCGTGTAAC | CGCCCGCCGAGCATTTCAACTA | 106 | 100 |
|  |  |  |  |  |  |
| MCRLD-MPLEX | *msr*(E) | AGCGGATGAACCAACCAGCCAC | TTGCATGCCCAAGTCGTCCAGC | 371 | 100 |
|  | *erm*(42) | GGCATTAAGCAAAATCTGCAAAGCCGT | AAGATGGTGCACCCGCATACTTT | 281 | 100 |
|  | *mph*(E) | TCACTTGCTGAAGCACACGGCT | CGACGAGGAATACGCAGCAACCA | 128 | 100 |

**Supplementary Table S4.** Percentage (No.) of *Mannheimia haemolytica* and *Pasteurella multocida* recovered from post-mortem bovine respiratory disease (BRD) mortalities by diagnosis

| **Species recovered from BRD mortality** | **Abscess** | **Arthritis** | **Bronchointerstitial pneumonia** | **Bronchopneumonia** | **Chronic pneumonia** | **Fibrinous pleuritis** | | **Fibrinous pneumonia** |
| --- | --- | --- | --- | --- | --- | --- | --- | --- |
| *M. haemolytica* | 1 (1) | 3 (5) | 0 (0) | 19 (28) | 10 (14) | | 0 (0) | 67 (99) |
| *P. multocida* | 0 (1) | 3 (5) | 1 (0) | 40 (28) | 27 (14) | | 1 (0) | 27 (19) |

| **Supplementary Table S5**. Percentage (no.) of *Mannheimia haemolytica* isolates from each serotype by study period. | | | |
| --- | --- | --- | --- |
|  | **Study 1**  **2011-2012** | **Study 2**  **2014-2015** | **Study 3**  **2015-2016** |
| Serovar 1 | 90 (36) | 96.25 (77) | 81.5 (22) |
| Serovar 2 | 2.5 (1) | 1.25 (1) | - |
| Serovar 6 | 7.5 (3) | 2.5 (2) | 18.5 (5) |
